# Glioblastoma Stem-Like Cells: Characteristics, Microenvironment, and Therapy

**DOI:** 10.3389/fphar.2016.00477

**Published:** 2016-12-07

**Authors:** Yang Yi, I-Yun Hsieh, Xiaojia Huang, Jie Li, Wei Zhao

**Affiliations:** ^1^Key Laboratory for Stem Cells and Tissue Engineering, Ministry of Education, Sun Yat-sen UniversityGuangzhou, China; ^2^Department of Histology and Embryology, Zhongshan School of Medicine, Sun Yat-sen UniversityGuangzhou, China; ^3^Department of Breast and Thyroid Surgery, The First Affiliated Hospital of Sun Yat-sen UniversityGuangzhou, China

**Keywords:** glioblastoma stem-like cells, tumor microenvironment, nanoparticle, epigenetic plasticity, nanocarrier technologies

## Abstract

Glioblastoma multiforme (GBM), grade IV astrocytoma, is the most fatal malignant primary brain tumor. GBM contains functional subsets of cells called glioblastoma stem-like cells (GSCs), which are radioresistant and chemoresistant and eventually lead to tumor recurrence. Recent studies showed that GSCs reside in particular tumor niches that are necessary to support their behavior. To successfully eradicate GBM growth and recurrence, new strategies selectively targeting GSCs and/or their microenvironmental niche should be designed. In this regard, here we focus on elucidating the molecular mechanisms that govern these GSC properties and on understanding the mechanism of the microenvironmental signals within the tumor mass. Moreover, to overcome the blood–brain barrier, which represents a critical limitation of GBM treatments, a new drug delivery system should be developed. Nanoparticles can be easily modified by different methods to facilitate delivery efficiency of chemotherapeutics, to enhance the accumulation within the tumors, and to promote the capacity for targeting the GSCs. Therefore, nanotechnology has become the most promising approach to GSC-targeting therapy. Additionally, we discussed the future of nanotechnology-based targeted therapy and point out the disadvantages that should be overcome.

## Introduction

Glioma is a type of tumor that arises from neoplastic glial cells. It makes up about 30% of all cases of brain and central nervous system tumors and 80% of all malignant brain tumors (Goodenberger and Jenkins, 2012). Glioblastoma multiforme (GBM), the most common and fatal form of a primary brain tumor, accounts for approximately 60% of all glioma cases and is categorized as grade IV glioma. GBM has a peak incidence in adults older than 40 years of age, with 2.96 cases per 100,000 people per annum in the USA (Ostrom et al., 2015). Local invasiveness, neoangiogenesis, and intratumor heterogeneity are among the most important hallmarks of the aggressiveness of GBM (Jain et al., 2007; Aum et al., 2014; Paw et al., 2015). Conventional GBMs can be subdivided into primary and secondary tumors on the basis of clinicopathologic stratification (Kleihues and Ohgaki, 1999). Since 2008, the Cancer Genome Atlas (TCGA) Research Network has generated a comprehensive catalog of genomic abnormalities driving tumorigenesis. With the help of this powerful tool, GBM is further categorized into four main subtypes: classical, mesenchymal, proneural, and neural type based on the mutational spectrum (Phillips et al., 2006; Verhaak et al., 2010). Considering the striking diversity among GBM subtypes and the clinical observation that patients do not switch between subtypes during various stages of this disease, different therapeutic strategies for each group of GBM patients may yield more effective outcomes.

The current standard treatment established by Stupp et al. (2005) involves concomitant administration of temozolomide with fractionated radiotherapy, with subsequent adjuvant temozolomide. Despite the treatment advances and increased understanding of the molecular and cellular mechanisms of GBM, current therapy is rarely curative due to the infiltrative nature of these tumors and their resistance to radiotherapy and chemotherapy (Jhanwar-Uniyal et al., 2015). Even after the maximal safe surgical resection, the residual tumor-initiating cells (TICs) that infiltrate the surrounding brain tissue could easily recapitulate the tumor and often become more aggressive (Dick, 2008). Owing to the near-universal tumor recurrence, patients with a diagnosis of GBM have the median survival period of only 12–15 months, with only 10% of the patients surviving 5 years (Stupp et al., 2009). These dismal outcomes reinforce the urgent need for novel therapeutic strategies to beat this devastating disease. By means of constant experiments and verification of the results, it is believed that nanomaterials may be excellent carriers for tumor therapy. According to the traits of nanomaterials, such as small particle size, large specific surface area, easy modification, quantum dynamical behavior, good permeability, and better solubility than traditional drugs, nanomaterials have become the promising drug carriers that are used for targeting the central neural system. In addition to the research into drug design, studies on characteristics of cancer cells and cancer stem cells (CSCs) should also help to improve the curative effect.

Compelling evidence suggests that CSCs play a major role during initiation, progression, and recurrence of a tumor and are primarily responsible for radiation and chemotherapy resistance and poor survival of GBM patients (Bradshaw et al., 2016). It is, therefore, not surprising that many therapeutic approaches have been devised to specifically target CSCs, but still with limited success (Seymour et al., 2015; Liebelt et al., 2016). The emerging nanotechnology may serve as a powerful tool to overcome the difficulties encountered with glioblastoma stem-like cell (GSC)-targeting treatments. By modification of nanomaterials researchers can effectively improve the capacity for transport into brain parenchyma and for GSC targeting. For instance, Kim et al. (2014) designed a cancer-targeting nanodelivery platform system, which can carry a variety of drugs and substances and can efficiently enhance the drug accumulation in GSCs.

Thus, in this review, we summarize the key features of GSCs and their microenvironmental niche. Then, we will discuss the application of nanotechnology to the development of GSC-targeting curative strategies and point out its future directions.

## GSCs and Their Characteristics

According to the CSC hypothesis, tumors are hierarchically organized and, at the apex of the hierarchy, are cells that display stem cell properties. These properties include (a) self-renewal, as well as the ability to differentiate into different cell lineages forming the complexity of the tumor; (b) multiple drug resistance and radiation resistance; (c) high tumorigenicity; (d) similar signaling pathways as normal stem cells. In addition to the above CSCs features, GSCs have the peculiarities of cloning neurosphere-like clusters of cells. The presence of GSCs was first demonstrated by the identification of a CD133^+^ cell subpopulation that is capable of tumor initiation *in vivo* (Singh et al., 2004). These tumor stem cells can form neurospheres *in vitro* and share many characteristics with stem cells such as the self-renewal ability and multipotent differentiation (Yuan et al., 2004). Moreover, one report shows that two CSC populations, which greatly differ in their growth properties and tumor-initiating ability, can reside within distinct regions of the same human GBM (Piccirillo et al., 2009). Therefore, chemotherapy may not exactly target the active subtype of CSC populations and thus have a poor curative effect. According to the above theory, Sugimori et al. (2015) hypothesized that in each cultured passage, heterogeneous clonal sublines of a glioma sphere-forming model should display gradually increased proliferative ability. But to their surprise, they found that the self-renewal of heterogeneous GSC populations is actually controlled by the power-law growth mechanism. The power-law growth theory may be a promising development in anticancer theories (Sugimori et al., 2015). Another latest study suggests that differentiated GBM cells can be fully reprogrammed into tumor stem-like cells by induction of only four core transcription factors (TFs)—POU3F2, SOX2, SALL2, and OLIG2, further supporting the GSC plasticity and tumor hierarchy existing within GBM (Suvà et al., 2014).

### Stem Cell Markers in GBM

CD133, a cell surface marker of normal neural stem cells (NSCs), is commonly used to distinguish GSCs (Singh et al., 2004; Calabrese et al., 2007). One report revealed that as few as 100 CD133^+^ cells are sufficient for the tumor initiation in the brains of immunodeficient mice, which was not the case for the CD133^-^ population (Singh et al., 2004). In addition, an increased proportion of CD133^+^ cells in GBM correlates with worse prognosis and poorer survival (Zeppernick et al., 2008; Metellus et al., 2011). The percentage of CD133^+^ cells is significantly higher in recurrent GBMs after radiotherapy and chemotherapy as compared with primary tumors (Tamura et al., 2013). The CD133 signature effectively separates GBM from lower-grade gliomas, and its enrichment has been attributed to the aggressiveness of the tumor (Yan et al., 2011). Moreover, CD133^+^ glioma cells, but not CD133^-^ cells, interact closely with vascular endothelial cells (ECs) in 3D Matrigel cultures to form a perivascular niche that promotes the initiation of brain tumors (Calabrese et al., 2007). Nonetheless, whether CD133 can serve as a sole CSC marker for GBM has been questioned after a series of subsequent papers. Wang et al. (2008a) demonstrated that CD133^-^ subpopulations also have the potential to initiate GBM tumor formation, and the xenograft tumors initiated by CD133^-^ cells are capable of producing CD133^+^ progeny *in vivo*. Another study proved the expression of nestin, glial fibrillary acidic protein (GFAP), and neuron-specific enolase (NSE) in CD133^-^ cells of GBM (Prestegarden et al., 2010). After further isolation of cells using different markers, the authors found that all these CD133^-^ subpopulations produce tumors without significant differences in survival or tumor take rates. On the other hand, there was a trend toward lower take rates for CD133^-^ GBM subpopulations expressing GFAP and NSE (Prestegarden et al., 2010). These observations suggest that the stemness of GBM cells may not be identified solely on the basis of CD133 expression. Apart from CD133, several other cell surface markers such as SSEA-1, CD44, integrin α6, L1CAM, and A2B5 have been used to enrich stem-like populations in GBM (Son et al., 2009; Anido et al., 2010; Lathia et al., 2010; Tchoghandjian et al., 2010; Cheng et al., 2011). The effectiveness of these various cell surface stem cell markers is still controversial.

Several key transcriptional factors involved in stem cell maintenance are also proved to be highly expressed in subpopulations of GSCs, such as c-Myc, SOX2, OCT4, NANOG, SALL4, STAT3, Bmi1, and KLF4. Wang et al. (2008b) showed that c-Myc is highly expressed in GSCs compared with non-stem glioma cells. Knockdown of c-Myc in GSCs reduced proliferation and increased apoptosis. But non-stem glioma cells did not dependent on c-Myc signaling for survival. SOX2, OCT4, and NANOG, which are critical in maintaining pluripotency in ESCs, are known to be highly expressed in subpopulations of GSCs, maintaining their self-renewal and cellular proliferation (Wang et al., 2008b). Current research demonstrates positive correlations between SOX2, OCT4, and NANOG expression and pathological grade of gliomas. Furthermore, Zbinden et al. (2010) showed that NANOG is preferentially expressed in GSCs modulating the tumorigenicity, proliferation and gliomasphere clonogenicity in GBM. By interacting with NANOG, SALL4 is highly expressed in gliomas than in normal brain tissue and correlates with a poor prognosis. Additionally, inhibition of SALL4 decreases cellular proliferation in gliomas and promotes apoptosis (Zbinden et al., 2010). But the use of SALL4 as a marker for GSCs is still controversial. Sherry et al. (2009) showed that STAT3 is a critical regulator of proliferation and maintenance of multipotency in GSCs. Recent reports further confirmed that loss of STAT3 suppressed tumorigenicity and enhanced radiosensitivity of GSCs. Similarly, Abdouh et al. (2009) showed that BMI1 is highly enriched in CD133-positive cells in GBM tumors. Later, Baxter et al. (2014) found silencing BMI1 eliminates tumor formation of pediatric glioma CD133^+^ cells. These findings highlighted the importance of BMI1 in GSCs. Zhu et al. (2014) showed that the PGI-KLF4 pathway regulates self-renewal of GSCs in human gliomas, suggesting blockade of the PGI-KLF4 pathway may provide a therapeutic strategy against GSCs. These studies reinforce the importance of deep understanding of GSC markers. Proper validation of these CSC markers will further refine the identification and characterization of the CSC population in GBM and facilitate successful treatment of malignancies.

### Implicated Signaling Pathways

The qualities of multipotency and self-renewal are maintained by the activation of a number of developmental signaling pathways such as Notch, Sonic hedgehog (SHH), and Wnt pathways, which are shared between NSCs and CSCs in GBM. Increasing evidence suggests that aberrations of these signaling pathways are implicated in the origin and maintenance of CSCs in GBM.

The Notch pathway regulates the cell-lineage decisions during embryogenesis and plays a critical role in the progression of tumorigenesis such as proliferation, angiogenesis and cell migration (Lasky and Wu, 2005; Leong and Karsan, 2006; Dufraine et al., 2008). Notch-1 and its ligands are overexpressed in many GBM cell lines and primary tumors and activation of this pathway contributes to CSC survival and proliferation (Purow et al., 2005; Kanamori et al., 2007). A recent study confirmed that Notch blockade combined with a standard-of-care treatment has an anti-CSC effect and provides an improved survival benefit for GBM patients as well as new insights for further clinical studies (Yahyanejad et al., 2016).

Activation of the SHH pathway drives the development of neural crest stem cells and the sympathetic nervous system (Wu et al., 2010). Thus, it is not surprising that dysregulation of the SHH pathway correlates with the central nervous system tumorigenesis (Dahmane et al., 2001). The SHH pathway is active in CSCs of GBM to maintain self-renewal and to induce tumorigenesis by regulating the expression of stemness genes (Clement et al., 2007). Inhibition of the SHH pathway by cyclopamine specifically eliminates the CSC population in GBM (Bar et al., 2007). Recently, Filbin et al. (2013) demonstrated that inhibition of phosphatidylinositol 3-kinase (PI3K) and SHH pathways synergistically reduces the growth of GBM *in vitro* and *in vivo*, indicating a novel therapeutic approach to GBM.

It is well-established that the Wnt/β-catenin signaling pathway plays crucial roles in the regulation of embryogenesis, homeostasis, regeneration, and stem cell pluripotency (Nelson and Nusse, 2004). Numerous studies have elucidated the regulatory connection between the Wnt/β-catenin signaling pathway and GSCs. One report shows that glioma oncogene *PLAGL2* promotes gliomagenesis and maintains the stemness of GSCs by activating the Wnt/β-catenin pathway (Zheng et al., 2010). Another study revealed the direct interaction between TF FoxM1 and β-catenin (Zhang et al., 2011). The FoxM1/β-catenin interaction controls the self-renewal of GSCs and is required for G2/M transition and proper mitotic progression. Further research confirmed the upregulation of master stem cell regulator SOX2 by FoxM1, which subsequently promotes the stemness and radioresistance of GBM (Lee et al., 2015). In addition, Jin et al. (2016) found that ectopic expression of inhibitor of differentiation 1 (ID1) suppresses the CULLIN3 E3 ubiquitin ligase and increases CYCLIN E protein stability in GBM. Loss of CULLIN3 simultaneously activates WNT and SHH signaling and thus promotes the GSC properties (Jin et al., 2016).

### Contribution of Epigenetic Alterations to GSCs

Aberrant epigenetic alterations are being increasingly recognized as a major factor contributing to the pathogenesis of many cancers, including GBM (Martinez et al., 2009; Kondo et al., 2014). Epigenetic silencing of the O^6^-methylguanine-DNA methyltransferase (MGMT) results in defective DNA repair and is associated with longer survival among GBM patients, indicating the significance of epigenetic mechanisms in GBM development (Hegi et al., 2005). Because the stem cell maintenance and differentiation require the precise modeling of chromatin, epigenetic alterations may confer a competitive advantage onto GSCs to adapt to the various requirements of their malignant state and to the genetic changes and environment. Stricker et al. (2013) demonstrated that resetting of DNA methylation by induced pluripotent stem cell reprogramming followed by lineage differentiation suppresses the malignant properties of GBM. In addition, comparative epigenomic analysis of chromatin maps revealed a module of developmental TFs that is coordinately activated in GSCs (Rheinbay et al., 2013). These TFs are essential for GSC maintenance and are normally epigenetically silenced, pointing to the existence of unique epigenetic regulatory programs in GSCs.

Polycomb group (PcG) proteins are a family of epigenetic regulators that regulate gene transcription by maintaining the repressive or active chromatin state. Dysregulation of PcG is thought to be closely related to the GSC maintenance and *in vivo* tumorigenicity (Li et al., 2013; Signaroldi et al., 2016). The polycomb repressor enhancer of zeste homolog 2 (EZH2) is upregulated in GBM and plays a crucial role in GSC maintenance (Suva et al., 2009). EZH2-dependent dysfunction of the bone morphogenetic protein (BMP) pathway contributes to the tumorigenicity of GSCs by both desensitizing GSCs to normal differentiation cues and by converting cytostatic signals to proproliferative signals (Lee et al., 2008). Another polycomb repressor, Bmi1, is also proven to be required for GBM development in an Ink4a/Arf-independent manner and regulates the differentiation capacity of GSCs (Bruggeman et al., 2007). Furthermore, very recent evidence shows that polycomb dysregulation in gliomagenesis affects transcriptional networks associated with invasiveness and dedifferentiation and indicates that ZFP423 is a master PcG target mediating the differentiation network (Signaroldi et al., 2016).

Some key developmental genes in GBM are also epigenetically altered to facilitate the activation of stem cell-like properties. Overexpression of the TF SOX2 has been reported in GBM and is mainly caused by aberrant DNA promoter demethylation. A knockdown of SOX2 in CSCs results in the loss of their self-renewal properties; this finding is suggestive of the importance of a pleiotropic role of SOX2 (Alonso et al., 2011). Another study showed that expression of HIPPO pathway transcriptional coactivator TAZ is silenced in lower-grade gliomas as well as proneural GBMs when compared with mesenchymal tumors. Functional analysis confirms that TAZ is a key modulator of mesenchymal differentiation in GBM whose activity is regulated epigenetically (Bhat et al., 2011). Nevertheless, at present, there is limited knowledge about the mechanisms via which epigenetic modifiers function in GSCs and the related therapeutic targeting.

## The Microenvironment of GSCS: The Tumor Vascular Niche

**FIGURE 1:**
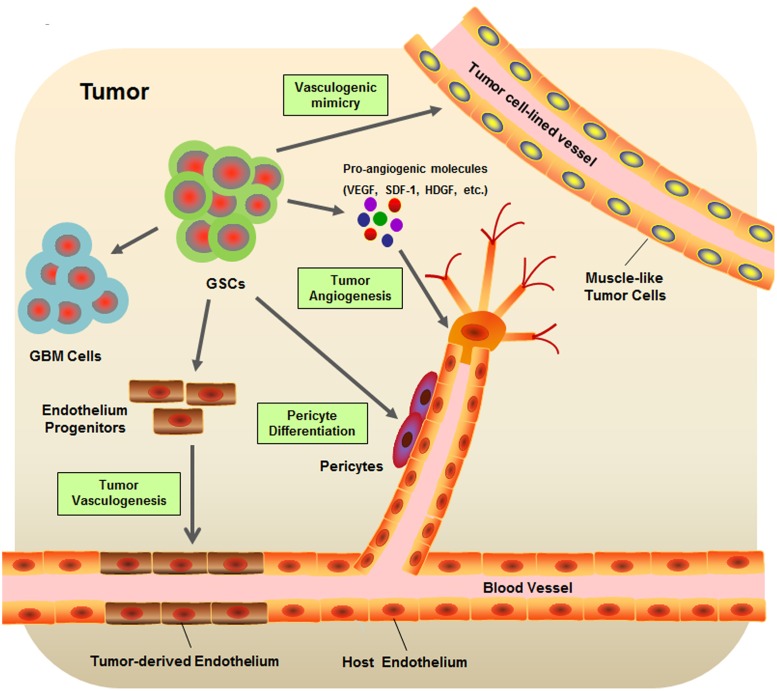
**Schematic diagram of glioblastoma stem-like cell (GSC) microenvironment.** GSCs are considered to be involved in many key events contribute to the formation of GBM vascular niche, including the tumor angiogenesis, tumor vasculogenesis, vasculogenic mimicry, and pericyte differentiation.

Cancer is an evolutionary and developmental process that is not only driven by genetic variations but also strongly shaped by numerous environmental factors. Accumulating evidence has shown that cancers are closely associated with their surrounding microenvironment consisting of neighboring cells, molecules, and vascular and lymphatic networks (Weinberg, 2008; Junttila and de Sauvage, 2013). As one of the most vascularized solid tumors, GBM orchestrates vascular niches to maintain self-renewal and survival of GSCs (Calabrese et al., 2007). In turn, GSCs may also regulate the tumor vasculature and subsequently promote the progression of tumor angiogenesis (Jhaveri et al., 2016). Many efforts have been devoted to unveiling the sophisticated interplay between GSCs and the tumor vascular niche (**Figure 1**).

### Tumor Vasculature in GBM

The human vasculature is a highly organized and dynamic system providing essential paths for the body to transport gasses, nutrients, waste products, or cells. In stark contrast to normal blood vessels, tumor vasculature, especially in GBM, forms a different network resulting in structural and functional abnormalities such as irregular architecture, high permeability, severe hypoxia, loss of hierarchy, and a compromised blood – brain barrier (Jain et al., 2007). Normal blood vessels are formed mainly by the mechanisms of vasculogenesis and angiogenesis (Risau, 1997). Vasculogenesis is the process of blood vessel formation occurring via *de novo* production of endothelial progenitor cells (EPCs) during organogenesis and fetal development, whereas angiogenesis represents the process of new vessel development from preexisting vasculature (Semenza, 2007). Construction of a primitive vascular network by vasculogenesis and the following angiogenesis that is responsible for expansion and remodeling of the existing vasculature implement the formation of both normal and GBM vasculature. In addition, two other types of neovascularization—vascular co-option and vasculogenic mimicry—specifically characterize the formation of GBM vasculature (Liebelt et al., 2016).

### Promotion of Tumor Angiogenesis by GSCs

The critical role of GSCs during glioma angiogenesis has been widely studied. Vascular endothelial growth factor (VEGF), one of the most important proangiogenic molecules, is significantly upregulated in the medium of CD133^+^ GSCs as compared to that of CD133^-^ GSCs. This high level of VEGF promotes human microvascular EC migration and tube formation (Bao et al., 2006). Further research suggested that GSCs contribute to tumor angiogenesis by promoting both local EC activity and systemic angiogenic processes involving bone marrow-derived EPCs in a VEGF-dependent and stroma-derived factor 1 (SDF-1)-dependent manner (Folkins et al., 2009). Expression of VEGF and SDF-1 could be stimulated in GSCs by hypoxia via activation of the PI3K/AKT signaling pathway (Heddleston et al., 2009; Ping et al., 2011). In addition, differential proteomic analysis indicates overexpression of another proangiogenic factor, hepatoma-derived growth factor (HDGF), in GSCs. Functional studies prove the role of HDGF in promoting migration of human cerebral ECs *in vitro* and in induction of neoangiogenesis *in vivo* (Thirant et al., 2012).

### Involvement of GSCs in Tumor Vasculogenesis via Transdifferentiation into Endothelial Cells

Vasculogenesis is a *de novo* vascular formation through the differentiation of EPCs during organogenesis and fetal development. Emerging lines of evidence suggest that, besides angiogenesis, tumor vascularization can also proceed via endothelial transdifferentiation of GSCs (Folkins et al., 2009; Ricci-Vitiani et al., 2010; Wang et al., 2010). Two independent groups reported that a subpopulation of ECs in GBM harbors the same genomic alterations as tumor cells, pointing to a neoplastic origin of the tumor vascular endothelium (Ricci-Vitiani et al., 2010; Wang et al., 2010). In particular, Wang et al. (2010) suggest that CD133^+^ GSCs can give rise to intermediate CD133^+^/CD144^+^ progenitor cells and subsequently differentiate into ECs with the upregulation of CD105, CD31, CD34, and VEGFR-2. Ricci-Vitiani et al. (2010) showed that CD133^+^ GSCs grown under endothelial conditions generate microvascular cultures of CD31^+^ and Tie2^+^ cells, and the vessels of a xenograft tumor in immunocompromised mice formed by GSCs are primarily composed of human CD31^+^ ECs. The presence of tumor-derived ECs originating from GSCs was then verified by Soda et al. (2011) using a genetically engineered mouse model of GBM. Although, the significance of tumor vasculogenesis during the development of GBM vasculature is still controversial as compared with tumor angiogenesis (Rodriguez et al., 2012), it is clear that GSCs may perform a critical function in both major mechanisms formation of tumor vasculature.

### GSCs Contribute to Vasculogenic Mimicry

In contrast to vasculogenesis, vasculogenic mimicry is defined as the ability to form fluid-conducing vessel-like networks by highly invasive tumor cells other than ECs (Chen and Chen, 2014). One study identifies the tubular type blood vessels formed by tumor cells in GBM tissues (El Hallani et al., 2010). Follow-up experiments suggest that CD133^+^ GSCs can transdifferentiate into smooth muscle-like cells that may constitute a part of the tumor cell-lined vessel wall as the essential muscular component. Yao et al. (2013) demonstrated the existence of non-EC-lined vessels in GSC-derived murine xenograft tumors and human primary GBM and further demonstrated that VEGF receptor 2 (VEGFR-2) is necessary for the formation of tubular structures by GSCs. Moreover, Mao et al. (2013) confirmed the contribution of CD144 to the vasculogenic mimicry of GSCs, especially under hypoxic conditions.

### GSCs Give Rise to Pericytes

Normal and tumor vessels share two distinct but interdependent cellular components: ECs and vascular mural cells embedded in the basement membrane of microvessels called pericytes (Sweeney et al., 2016). Depletion of pericytes in tumor vasculature suppresses tumor growth but enhances epithelial-to-mesenchymal transition (EMT) and metastasis; these observations are suggestive of the essential role of pericytes in balancing cancer progression and metastasis (Cooke et al., 2012). Emerging evidence shows that GSCs can give rise to pericytes to support vessel function and facilitate tumor growth (Cheng et al., 2013; Guichet et al., 2015). Cheng et al. (2013) suggest that pericytes are commonly derived from neoplastic cells in GBM. Selective elimination of pericytes by ganciclovir disrupts tumor vessels and inhibits tumor growth. Functional studies revealed that GSCs are recruited toward ECs via the SDF-1/CXCR4 axis, and that the differentiation of GSCs into pericytes is induced by TGF-β secretion (Cheng et al., 2013). A recent study showed that Notch-1 stimulation triggers the expression of pericyte cell markers in GSCs and generates highly vascularized and poorly disseminating graft tumors containing GBM-derived pericyte-like cells (Guichet et al., 2015). Moreover, TF HMGA2 was found to be expressed in both GSCs and pericytes of GBM. Depletion of HMGA2 in GSCs abrogates their potential for pericyte differentiation, indicating its role in self-renewal properties of GSCs (Zhong et al., 2016).

## Glioblastoma Therapy

The traditional treatment program of malignant GBM is surgery, radiotherapy, chemotherapy, and traditional Chinese medicine. Because the growth characteristic of GBM is invasive growth, there are no clear boundaries distinguishing the lesions from the normal brain parenchyma. Surgical treatment cannot eliminate the lesions. Residual cancer cells can lead to relapse. Thus, surgery has limitations. In addition, there are limitations of radiotherapy and chemotherapy respectively. Combining both treatment strategies is more often used in GBM. Even so, therapeutic benefits are still difficult to improve. Novel treatments are urgently needed. Nanotechnology has several advantageous characteristics that can be used in tumor therapy. In the following subsection, we are going to expound the application of nanotechnology to GBM management.

Nanoparticles are composed of artificial materials or natural polymers, usually ranging in the grain diameter from 1 to 100 nm (Gabathuler, 2010). At present, most clinical nano-drug diameters are approximately 10–1000 nm (Chen and Liu, 2012). As compared to traditional chemotherapeutics, nanoparticles have a much smaller grain diameter, larger specific surface area, stronger adhesion, easier permeation through biological barriers, better lipid solubility, and an accurate targeting ability. Nanoparticles gradually assume a substantial role in tumor therapy and targeted treatment of CSCs. Nanoparticles can be modified by means of a variety of materials, which, for example, can inhibit CSCs growth and renewal (Wang et al., 2011), increase their biological barrier permeability, and improve targeting of treatment. Modified nanoparticles effectively enhance the transportation in the brain (Woodworth et al., 2014; Kim et al., 2015).

## Transport of Nanoparticles to the Brain

Drug delivery to the brain takes place via two pathways: one way is intravenous injection that streams the drug to intracerebral vessels and transfers them through the blood–brain barrier (BBB) to brain parenchyma. Composed of tight junctions between brain capillary ECs, the BBB maintains the stable chemical microenvironment of the cerebral periphery and prevents harmful substances from invading the brain. The robust physical barrier ensures that 98% of small molecules and whole macromolecules cannot pass through (Wei et al., 2014). Meanwhile, the tight junction prevents the transmembrane transport via paracellular route (Pardridge, 2006; Wong et al., 2013).

The other way involves extravascular systems: cerebrospinal fluid (CSF) and interstitial fluid (ISF).

The extravascular system of CSF is well-known. There is ∼100–150 ml if CSF in human spinal canal circulating every 4–5 h. CSF flows back into blood through arachnoid granulation near the sagittal sinus that forms CSF and blood circulation. Quantitative resistance ensures that is difficult for macromolecular material to permeate through the arachnoid granulation. We call the resistant entity the “blood–cerebrospinal fluid barrier (BCSFB).” The solute exchange rate is much slower in BCSFB than in BBB. As the result, the method of CSF drug injection is rarely used for treatment of brain tumors (Pardridge, 2006). As the tumor grows, the BBB will be broken down and increase its permeability. Tumor ECs help to develop new vessels stretching into the tumor, yet the new vascular epithelial cell junction is not tight enough. Because of the loose vessel endothelium and a lack of lymphatics in tumor parenchyma, it is easier for substances to flow into tumor parenchyma than to flow out. This phenomenon is called solid tumor enhanced permeability and retention effect. Some investigators believe that particle diameter greater than 100 nm makes it more difficult for materials to pass through capillary walls; however, the grain diameter less than 20 nm will make it easier for a material to return to blood circulation. Thus, these researchers suggest that particle diameters greater than 100 nm and less than 20 nm are the best sizes for effective accumulation of a substance in tumor tissue (Perrault et al., 2009).

Another extravascular system is the ISF. ISF is composed of two parts: hydrostatic pressure and the colloid osmotic pressure. The high interstitial fluid pressure (IFP) has been proved to be a crucial barrier of drug delivery (Jain, 1987). Most solid tumors have an increased IFP due to high vessel permeability, poor perfusion, and high cell density around the blood vessels. High IFP contributes to a low transcapillary transport in tumors, therefore it is responsible for the decreased intake of drugs. There are several types of regents to reduce tumor IFP in animal models and patients, such as anti-angiogenic drugs (VEGF inhibitors or PDGF inhibitors), TGF-beta inhibitors, Dexamethasone, Bradykinin agonists, Nicotinamide, and pro-inflammatory factor PGE1 (Heldin et al., 2004). In addition, some chemotherapeutics, such as carboplatin and paclitaxel, can enhance the effectiveness of anti-angiogenic drugs. Heist et al. (2015) showed that combination of anti-VEGF and chemotherapy improved the overall survival and the tumor free period of patients with metastatic non-small lung cancer. Zhou et al. (2008) found that VEGFR inhibitor, sunitinib, enhanced tumor distribution of temozolomide due to vascular normalization and stability and reduction in IFP. However, Ribatti criticized that the vessel normalization followed by normalization of permeability may become the obstacle to chemotherapy (Ribatti, 2011). It might be true, but we think that a better understanding of IFP mechanisms, and combination of chemo-drugs, vascular normalization drugs, and the nanoparticle delivery system to modulate IFP, may represent the new strategy of glioma therapy.

**FIGURE 2:**
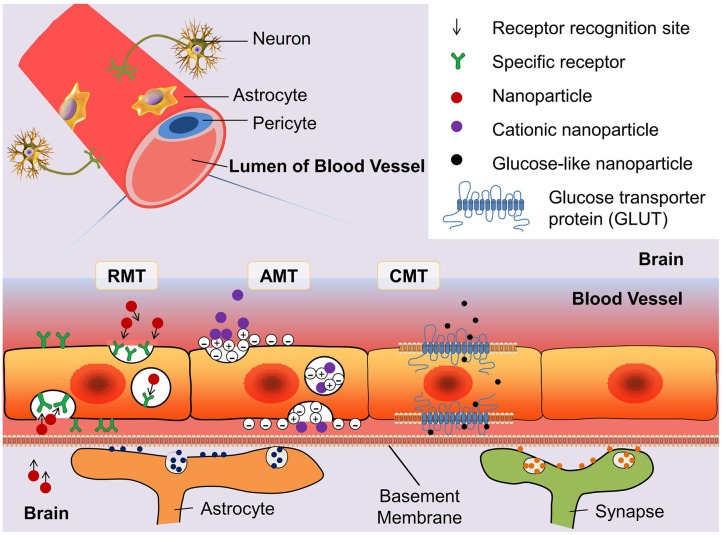
**Schematic diagram of three blood–brain barrier (BBB) transport routes: receptor-mediated transportation (RMT), adsorptive-mediated transportation (AMT), and carrier-mediated transportation (CMT).** The glucose transporter protein (GLUT) is used to represent the structure of CMT. By modifying the structure of nanoparticles can enhance the binding ability of drugs with the transporter.

A few soluble plasma molecules can be transported across the BBB by the methods of bulk-phase/fluid-phase transcytosis (FMT). This is because the cerebral epithelial cells possess high-density clathrin-coated pits/vesicles that prevent negatively charged ligands from FMT. Thus, only a few plasma proteins such as albumin and immunoglobulin G transferrin can randomly move across the BBB by FMT. According to the characterization of clathrin-coated pits/vesicles, parts of molecules can be transported by sinking into the clathrin-coated cells membrane (Herve et al., 2008). This process can be classified as receptor-mediated transcytosis (RMT) and adsorption-mediated transcytosis (AMT) depending on the transport mechanism. Nanoparticles can also be transported across the BBB by means of the carrier-mediated transportation (CMT), which usually transports big-molecule nutrients such as glucose and acids (**Figure 2**). There are other methods such as the paracellular aqueous pathway, transcellular lipophilic pathway, cell-mediated transcytosis, and efflux pumps (Chen and Liu, 2012). Nonetheless, the efficiency of transport policies is not satisfactory. How to break the transport limitations and design a novel system for conveyance is a new challenge. In the following subsection, we highlight the mechanisms of RMT, AMT, and CMT that are used frequently for penetration of the BBB.

### Receptor-Mediated Transportation

Receptor-mediated transcytosis relies on specific receptors on the BBB that transport endogenous polymers depending on the hydrolysis of ATP. It means that RMT has high specificity, affinity, and energy dependence. Herve et al. (2008) indicated that there are only a few peptides and proteins can be transported through the BBB, e.g., insulin, insulin-like growth factors, iron-transferrin, low density lipoprotein, and amyloid β proteins. In recent years, more and more researchers report that large molecules can pass through the RMT into the brain. Research into transferrin receptor shows that The polyester poly (D,L-lactide-co-glycolide) nanoparticles coated with transferrin can improve the adhesion to the cell surface and endocytosis (Chang et al., 2009). Otherwise, carbon nanospheres, large particles ranging from 100 to 500 nm, were confirmed to cross the BBB by clathrin-mediated endocytosis (Selvi et al., 2012).

### Adsorptive-Mediated Transcytosis

The AMT mechanism is a non-specific process that does not depend on the specific binding sites. Thus, the number of receptor molecules has no influence on the capacity of AMT. Transport is achieved by mutual attraction between a positive charge on nanoparticles and a negative charge on BBB EC membranes. Nonetheless, AMT has several undesirable features: low affinity, a lack of specificity, and poor targeting ability. Those characteristics limit practical applications of AMT. AMT can transport only cationic proteins or cell-penetrating peptides (Wei et al., 2014), e.g., antibody fragments, albumin, histone, or protamine (Herve et al., 2008). Nevertheless, those materials can combine with a variety of cargos, whereas nanoparticles may slightly promote the drug delivery capacity of AMT. Lu et al. (2005) discovered bovine serum albumin conjugated with poly(ethyleneglycol)-poly(lactide) nanoparticles (BSA-NPs), which cross the BBB by AMT with 7.76-fold higher permeability after cationization (CBSA-NP)). The following research indicates that the factor influencing the crossing of the BBB by CBSA-NPs through AMT may be related to the surface CBSA density of the particles. In addition, the mechanism of CBSA-NP crossing the BBB through AMT is believed to involve initial connection with the negative charge on the brain capillary epithelial cells (Lu et al., 2007). These discoveries increased the suitability of AMT for transport across the BBB.

### Carrier-Mediated Transportation (CMT)

Blood–brain barrier ECs have been shown to have multiple carrier proteins, which can transport glucose, amino acids, nucleic acids, and other necessary nutrients across blood capillary vessels to brain tissue. CMT has high substrate specificity where those carriers bond only with particular endogenous substances. Nanoparticles should simulate the designated substance that can be transported across the BBB by CMT. The most widely recognized carriers are glucose transporter proteins (GLUTs). GLUT1 and GLUT3 are abundant in the mammalian neurocyte membrane and brain capillary epithelium membrane (McEwen and Reagan, 2004). Especially, GLUT1 is the major glucose transporter in the mammalian brain. Qin et al. (2010) designed a system to load glucose on the liposome surface that takes advantage of the transport by GLUT1. Jiang et al. (2014) tested 2-deoxy-D-glucose modified poly(ethylene glycol)-co-poly(trimethylene carbonate) nanoparticles (D-Glu-NP) loaded with paclitaxel (PTX) and found that they have a greater ability for transport across the BBB and less cytotoxicity than non-glucosylated nanoparticles which are also loaded with PTX. Because of the potential to increase penetration of the BBB via GLUT-mediated transcytosis and the drug accumulative capacity in GBM cancer cells via GLUT-mediated endocytosis, D-Glu-NP became a promising targeting delivery system (Jiang et al., 2014).

## Categories of Nanoparticles

The ideal nanodrug should possess several merits: (a) high efficiency of carrying drugs and generation of sufficient concentration in the target cells, (b) low cytotoxicity and immunogenicity (to be as harmless as possible for normal somatic cells), (c) few side effects (to increase patients’ quality of life), (d) stability in blood and prolonged blood circulation time (Chen and Liu, 2012), and (f) low cost (should also be expected). In the following subsection, we introduce four kinds of nanoparticles, which are frequently used in conjunction with varied chemotherapeutics, are reported to target GBM CSCs (**Table 1**). By means of different modifications, nanoparticles’ ability to cross the BBB can be significantly enhanced. As a result of nanoparticles’ enveloping the chemotherapeutics before the drug is delivered to GBM, this approach may prevent normal cells from being damaged and promote the targeting to GBM CSCs.

**Table 1:** The categories of nanotechnology application of GSCs.

Type	Structure	Characteristics	Decoration	Reference
Liposome	Membrane-like double molecular phospholipids	(a) Diameter ranged from 20 nm to 100 um	(a) scL	Kim et al., 2014
		(b) Amphiphilic, biocompatible, non-toxicity, and biodegradable		
Polymeric Nanoparticle	A kind of solid colloid particles created from polymer materials	(a) Diameter ranged between 1 and 1000 nm	(a) PEG	Cho et al., 2008
		(b) Water-soluble, non-toxic, biodegradable	(b) Cationic albumin-conjugated PEG	Lu et al., 2006
		(c) Easy-modified, drugs easy-combined	(c) PU-PEI-mi R145	Yang et al., 2012
Gold Nanoparticle	Nanoscale metal particles	(a) Properties of metal	(a) L-aspartate-TEM	Orza et al., 2013
		(b) Easy-modified		
Nano-carbon particle	Combined by graphene sheets	(a) Great electrical properties	(a) chitosan-CD133 monoclonal antibodies	Wang et al., 2011
		(b) Large specific area		
		(c) Ion adsorption		
		(d) Potential for gene delivery and detection of biological molecules		


*scL, a tumor-targeting nano-delivery platform discovered by Kim et al. (2014); PEG, polyethylene glycol; PU-PEI, cationic polyurethane with short branched chain polymer of polyethylene imine; TEM, temozolomide.*

### Liposomes

Liposomes are composed of a molecular phospholipid, which forms the membrane-like double molecular phospholipids structure. The diameter ranges from 20 nm to 100 μm. Traits of great biocompatibility, non-toxicity, and biodegradability made it a good carrier.

Kim’s research uncovered a tumor-targeting nanodelivery platform called scL: it can carry diverse carriers and systematically manage molecular drugs. As confirmed by experiments on colonic cells (HT-29, HCT-116) and glioma cells (U87, U251), liposomes may accumulate not only in non-cancer stem cells: cancer stem-like cells also have efficient cumulative effects. When liposomes carry *wtp53* genes, these particles may induce the death of cancer stem-like cells and growth inhibition. This discovery potentially enhances the ability to prevent relapse (Kim et al., 2014).

### Polymeric Nanoparticles

These are kinds of solid colloid particles created from polymer materials. The diameter ranges between 1 and 1000 nm. The most popular application is linkage of polyethylene glycol (PEG) to nanoparticle surfaces.

Lu et al. (2006) used cationic albumin-conjugated pegylated nanoparticles (CBSA-NP) merged with plasmid pORF-hTRAIL (pDNA) to perform non-viral gene therapy of cerebral glioma. *In vitro* experiment, CBSA-NP-hTRAIL been observed that after being transfected the C6 glioma cell they’re widely existed in the cytoplasm. Lysosome in the cytoplasm gradually removed the CBSA-NP and released the pDNA simultaneously. pDNA joined the nuclei and induced apoptosis at 48 h after transfection. They, then, intravenous injected CBSA-NP-hTRAIL into mice bearing C6 glioma. *In vivo* experiment verified CBSA-NP-hTRAIL can efficiently accumulate in the glioma cells, instead of normal brain cells. Lu’s study showed us that CBSA-NP-hTRAIL, which can promote apoptosis and inhibit the tumor cell growth, may be a potential non-invasive gene therapy of GBM (Lu et al., 2006).

**FIGURE 3:**
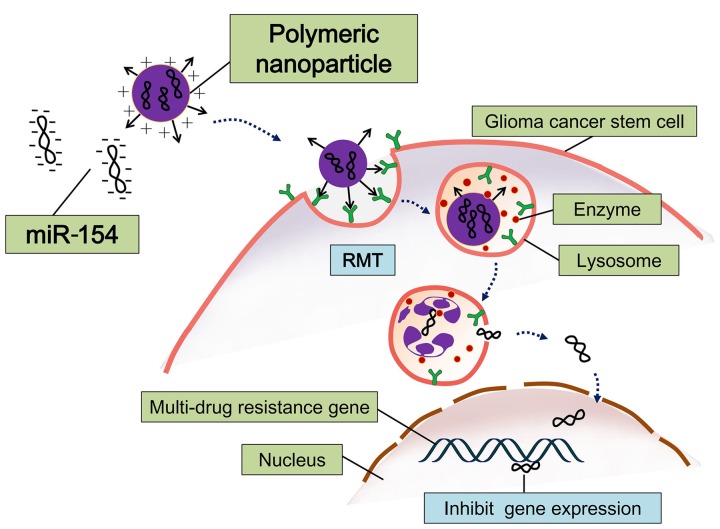
**Schematic diagram of polymeric nanoparticles loading with microRNA-145 (miR-145).** This miRNA-based therapeutic strategy can efficiently inhibit expression of multidrug resistance gene and anti-apoptotic gene in cerebral GSCs (Tyagi et al., 2016).

Yang used cationic polyurethane with short branched chain polymer of polyethylene imine (PU-PEI) as gene carriers, which carried microRNA-145 (miR145): this is called PU-PEI-miR145 (**Figure 3**). This construct can efficiently inhibit expression of multidrug resistance gene and anti-apoptotic gene in cerebral glioma cancer stem-like cells, thereby increasing sensitivity to radiotherapy and chemotherapy. Their research showed that PU-PEI-miR145 can significantly reduce the tumorigenic ability of malignant glioma CSCs in immunodeficient mice. Immunodeficient mice were injected *in situ* with glioblastoma and treated with the synergistic method of radiotherapy and chemotherapy; this approach noticeably increased their survival rate (Yang et al., 2012).

### Gold Nanoparticles (GNPs)

The use of gold nanoparticles to kill tumor cells has been widely reported, but applications aimed at killing tumor stem-like cells are rare. Orza et al. (2013) reported a kind of a nanogold carrier that, after loading with chemotherapy drugs, can selectively kill glioma CSCs and promote uptake of the drug by the CSCs. They used the gold nanoparticles treated with L-aspartate (GNP-L-aspartate), which is a triangular-structure ligand. The mean diameter was found to be ∼55 nm, and this aggregate can lengthen to become a chain ranging from 100 to 600 nm, and can be combined with temozolomide (GNP-L-aspartate-TEM). They found that there are some strong electrostatic interactions between GNP-L-aspartate and TEM that make the structure stable. Their research indicates that GNP-L-aspartate-TEM can induce an apoptosis mechanism in almost 90% of high-grade glioma-derived CSCs. This efficiency is better than that of treatment with TEM alone (apoptosis in 42% of CSCs). In addition, GNP-L-aspartate-TEM has higher bioactivity, lower toxicity, and better capacity for targeting glioma-derived CSCs (Orza et al., 2013).

### Nanocarbon Particles

Assembled from graphene sheets, these nanoparticles can be in several forms, e.g., carbon nanotubes or carbon nanospheres. Carbon nanotubes have the potential for gene delivery and detection of biological molecules (Cho et al., 2008). CD133 is recognized as the most validated malignant glioma CSC marker (Singh et al., 2004). Attachment of CD133 monoclonal antibodies onto carbon nanotubes that are modified by chitosan results in the micelles with an average diameter of ∼233 nm. Wang’s research confirmed that by means of CD133 antibodies, nanotubes can target glioblastoma CD133^+^ cells (GBM-CD133^+^). They mixed the micelles and malignant glioma cells and incubated them for some time, and then proceeded to 808-nm laser radiotherapy. The results revealed that GBM-CD133^+^ were eliminated clearly; however, GBM-CD133^-^ remained active. Those researchers subcutaneously injected the GBM-CD133^+^ which underwent radiotherapy as mentioned above into mice. Tumorigenicity and proliferation of GBM-CD133^+^ was reduced (Wang et al., 2011).

## Future Directions

For most chemical drugs, it is difficult to permeate the BBB. Even though the chemotherapeutics cross the BBB, a large wasteful dose is needed during the delivery process. This drawback may decrease the curative efficiency. Furthermore, chemotherapeutics have strong cytotoxicity: they not only damage cancer cells and CSCs but also normal somatic cells. The use of nanoparticles to carry the chemotherapeutics is a promising method that improves their delivery. Nanoparticles have such advantages as small grain diameter, large specific surface area, low toxicity, high affinity, ease of modification, and the ability to carry multiple cargoes. Encapsulation and chemical coupling technologies may facilitate the transport across the BBB, promote targeting capacity of varied drugs, and facilitate tumor diagnosis. There are more and more modification methods for nanoparticles in the literature. Yet, there are still a lot of unanswered questions:

(i) Selectivity of targeting to the brain tumor. This is a long-standing question. To enhance the drug efficiency, we expect a nanomedicine to successfully target the brain. Nonetheless, most nanoparticles are non-specific, which means they will also be delivered to other organs. This situation makes accurate targeting to the brain cancer cells or brain CSCs more difficult. Chen and Liu proposed using multiple targeting ligands, which are selected according to the pathological conditions of the disease and may thus successfully solve these problems (Chen and Liu, 2012). Yao et al. (2015) made some progress in tumor diagnosis techniques. Nanotheranostics is an emerging subject that often involves the diagnosis by means of magnetic nanoparticles, which can be imaged via magnetic resonance and hold great promise for beaconing the tumor position. Current research shows that magnetic nanoparticles are not only beacons but also promising drug delivery agents. They can target a brain tumor, control the release of drugs, and induce hyperthermia (Yao et al., 2015).
(ii) Individualized therapy. Because each individual has different receptor sensitivity, despite being given the same dose of drugs, the pharmacodynamics will differ among people. This notion highlights the importance of personalized treatment.
(iii) How to monitor the accumulation of nanodrugs in a brain tumor? There are many studies addressing this question and showing that the use of the iconography technology can detect the nanodrug at the nanoscale (Pohlmann et al., 2015).
(iv) Toxicity. Past and present studies have usually been focused on the reduction in cytotoxicity of the chemotherapeutics and often overlooked the issues with safety of the nanoparticles or nanodrugs. When nanoparticles are used in a treatment strategy, they should be rigorously analyzed as a clinical medication. Yet, there is no reference standard of toxicity. It is important to establish a set of standardized testing strategies for the safety assessment of nanodrugs (Elsaesser and Howard, 2012; Nystrom and Fadeel, 2012). Besides, the biological and toxicological behavior mostly depends on how the nanoparticles come into contact with a nanoscale biological structure. Elucidation of the laws governing the behavior of a bio-nano-interface *in vivo* may expand the practical applications of nanomedicine (Nystrom and Fadeel, 2012). Therefore, we look forward to the new knowledge that will be generated by researchers in the field of nanomaterials.

## Author Contributions

YY and I-YH summarized the literature and wrote the manuscript. XH drew the figures and revised the manuscript. JL and WZ supervised all the work and finalized the manuscript.

## Conflict of Interest Statement

The authors declare that the research was conducted in the absence of any commercial or financial relationships that could be construed as a potential conflict of interest.
